# Escaping through virtual gaming—what is the association with emotional, social, and mental health? A systematic review

**DOI:** 10.3389/fpsyt.2023.1257685

**Published:** 2023-11-08

**Authors:** Lucas M. Marques, Pedro M. Uchida, Felipe O. Aguiar, Gabriel Kadri, Raphael I. M. Santos, Sara P. Barbosa

**Affiliations:** ^1^Faculdade de Medicina, Instituto de Medicina Fisica e Reabilitacao, Hospital das Clinicas HCFMUSP, Universidade de São Paulo, São Paulo, São Paulo, Brazil; ^2^Pontifícia Universidade Católica de Campinas, Campinas, São Paulo, Brazil; ^3^Department of Psychiatry, Santa Casa of São Paulo Medical Science School, São Paulo, São Paulo, Brazil

**Keywords:** escapism, virtual games, video-game, E-sport, emotion regulation, mental health, health promotion, gamification

## Abstract

**Background:**

The realm of virtual games, video games, and e-sports has witnessed remarkable and substantial growth, captivating a diverse and global audience. However, some studies indicate that this surge is often linked to a desire to escape from real life, a phenomenon known as escapism. Much like substance abuse, escapism has been identified as a significant motivator, leading to adverse outcomes, including addiction. Therefore, it is crucial to comprehend the existing research on the connection between escapism and engagement in virtual gaming. This understanding can shed light on the reasons behind such practices and their potential impact on mental and public health.

**Purpose:**

The objective of this systematic review is investigate the findings pertaining to association between escapism and the practice of virtual games, such as video-games and e-sport.

**Methods:**

PUBMED and SCOPUS database were systematically searched. Six independent researchers screened articles for relevance. We extracted data regarding escapism-related measures, emotional/mental health-related measures and demographic information relevant to the review purpose.

**Results:**

The search yielded 357 articles, 36 were included. Results showed that: (i) Escapist motivation (EM) is one of the main motives for playing virtual games; (ii) EM is related to negative clinical traits; (iii) EM predicts negative psychological/emotional/mental health outcomes; (iv) EM is associated with impaired/negative perception of the real-world life; (v) EM predicts non-adaptive real social life; and (vi) EM is associated with dysfunctional gaming practices in some cases. However, EM can have beneficial effects, fostering confidence, determination, a sense of belonging in virtual communities, and representation through avatars. Furthermore, the reviewed findings suggest that EM was positively linked to mitigating loneliness in anxious individuals and promoting social activities that preserved mental health among typical individuals during the pandemic.

**Conclusion:**

Our review reinforces the evidence linking EM in the context of virtual games to poor mental health and non-adaptive social behavior. The ensuing discussion explores the intricate connection between escapism and mental health, alongside examining the broad implications of virtual gaming practices on underlying motivations for escapism in the realms of social cognition, health promotion, and public health.

## Introduction

It is well-established how games and virtual reality have become integral parts of our lives. Similarly, the increasing number of games being used for treatment or interventions to aid individuals with neurological disabilities (1), neurocognitive disorders (2), or to improve physical health (3) has gained prominence.

In the USA, a country with one of the largest numbers of players globally, approximately 97% of children and adolescents spend at least one hour playing video games daily (4). Motivations for playing include stress relief, the challenge of games, social interaction, with older adults more likely to cite cognitive benefits (5). Additionally, other studies have highlighted reasons such as escapism, achievement, and competition (6, 7).

As presented by Zanetta et al. (7), typically 10 motivations can be observed among online gaming players: (i) Advancement: Becoming powerful; (ii) Mechanics: How interested are you in the precise numbers and percentages underlying the game mechanics?; (iii) Competition: Doing things that annoy other players; (iv) Socializing: Getting to know other players; (v) Relationship: How often do you talk to your online friends about your personal issues?; (vi) Teamwork: Would you rather be grouped or soloing?; (vii) Discovery: How much do you enjoy exploring the world just for the sake of exploring it?; (viii) Role-Playing: How often do you role-play your character?; (ix) Customization: How important is it to you that your character’s armor/outfit matches in color and style?; and (x) Escapism: Escaping from the real world.

In the field of game studies, one term that has garnered significant interest is ‘escapism,’ which can be defined as the pursuit of escaping from real life into another fictional world (8). Recent research has sought to associate gaming motives with narcissism through escapism (9), coping strategies with negative outcomes related to escapism (10), gaming escapism as a factor for Internet Gaming Disorder (IGD) development (11), the moderating effect of escapism on the relationship between loneliness and negative outcomes (12), difficulties in emotion regulation (13), and the motive of competition as a strong predictor of IGD (14). Additionally, this phenomenon of escapism is also observed in other areas of life, such as addiction to alcohol (15, 16), dance (17), pornography (18) and social networks (19).

Lastly, recent research has indicated that gaming escapism can be also associated with excessive gambling (20) and addictive gaming behavior (21). Furthermore, it has been observed that negative outcomes of gaming, influenced by escapism motives, may be exacerbated by factors such as social anxiety (22) a competitive background (14), existing psychiatric distress (23) and low self-concept clarity (24).

It is essential to emphasize that escapism, when applied to gaming, does not inherently carry a negative connotation. It can also serve as an emotional regulation strategy, encompassing attentional distraction during emotionally intense situations and emotional suppression following an already established emotional impact (25). Moreover, restricting our understanding of escapism in gaming to its negative connotation can unfairly characterize gaming solely as a negative activity. However, gaming is not exclusively an act of escapism but can also be motivated by a quest for enjoyment and diversion (8).

Hence, to comprehensively assess the positive or negative nature of the association between Escapist Motivation (EM) and mental health, it is essential to consider various factors, including the cultural context in which gaming practices occur (26).

Considering the abundance of studies that have explored the relationship between escapism and the engagement in virtual games, it is now opportune to examine the emotional, social, and mental health impacts arising from gaming practices driven by escapism motives. Therefore, this systematic review aims to consolidate key studies linking virtual gaming practices with escapism, with the intent of evaluating whether escapism indeed serves as a determinant of such practices and whether these activities have a positive or negative effect on mental health. As previously mentioned, among various motivators for online gaming, we have chosen to focus on Escapism in this study. Unlike other potential motivators that often exhibit adaptive behaviors and are associated with positive orientations, such as the pursuit of novelty, building new relationships, and curiosity, Escapism appears to be characterized by a desire to distance oneself from reality. This tendency may stem from a limited emotional and psychological toolkit for coping with the real world.

In this context, drawing from existing literature, we anticipate discovering negative correlations between Escapist Motivation (EM) and aspects conducive to effective social adaptation, such as the cultivation of social skills, emotional regulation abilities, and overall well-being. Conversely, addiction-related behaviors like Internet Gaming Disorder (IGD), social withdrawal, feelings of loneliness, heightened stress, as well as elevated levels of anxiety and depression, are expected to exhibit a positive association with EM. These are, however, hypotheses that underpin the rationale for this systematic review, which aims to unveil various novel facets integral to the ongoing discourse on this subject.

## Methods

### Literature search

We conducted a comprehensive systematic search in the SCOPUS and PubMed databases using specific keywords: ‘Escapism,’ AND ‘Virtual game,’ OR ‘Virtual-game,’ OR ‘Video-game,’ OR ‘Video-game,’ OR ‘E-sport,’ OR ‘Esport.’ These keywords were applied to identify relevant articles with these terms in their titles, abstracts, or keywords. The search was conducted from May 10th to May 12th, 2023, without additional filters, such as publication year. Additionally, a manual search was performed to identify potential articles through references cited within selected articles.

### Literature selection: inclusion and exclusion criteria

We included all original studies that explored the potential correlation between Escapism and the practice of virtual games. These encompassed studies considering Escapism as a motive or motivator for gaming, as well as those assessing the use of Escapism as a coping strategy resulting from gaming practices. Eligibility for inclusion was limited to articles written in English. Consequently, articles with the following characteristics were excluded: (i) perspective articles, (ii) case reports, (iii) study protocols, (iv) review articles, (v) conference articles, (vi) non-emotional/social/mental health related measures, and (vii) articles written in languages other than English.

To ensure accuracy and consistency in the study selection process, duplicated records were removed, and six authors independently screened all titles and abstracts using the predetermined framework and selection criteria. Following the title and abstract selection phase, the full texts of the selected articles were obtained and analyzed. Any discrepancies were resolved through consensus among all authors.

### Data extraction

Following a comprehensive examination of the articles, the authors meticulously identified and gathered the most pertinent data concerning the association between Escapism and the practice of virtual games. The data collection and review procedures were independently carried out by six authors. To extract essential information needed to define the connection between Escapism and virtual game practice, a structured list of variables was employed. The variables were specifically tailored to capture crucial aspects relevant to this association:

*Sample and experimental design*: (i) Sample size; (ii) Country (iii) Characteristics that define the studied group; (iv) Adopted study design.

*Escapism and emotional measures*: (i) Escapism-related measures; (ii) emotional/social/mental health-related measures.

*Observed effect*: (i) Results observed associating Escapism and emotional/social/mental health aspects.

## Results

### Study retrieval

**Figure 1:**
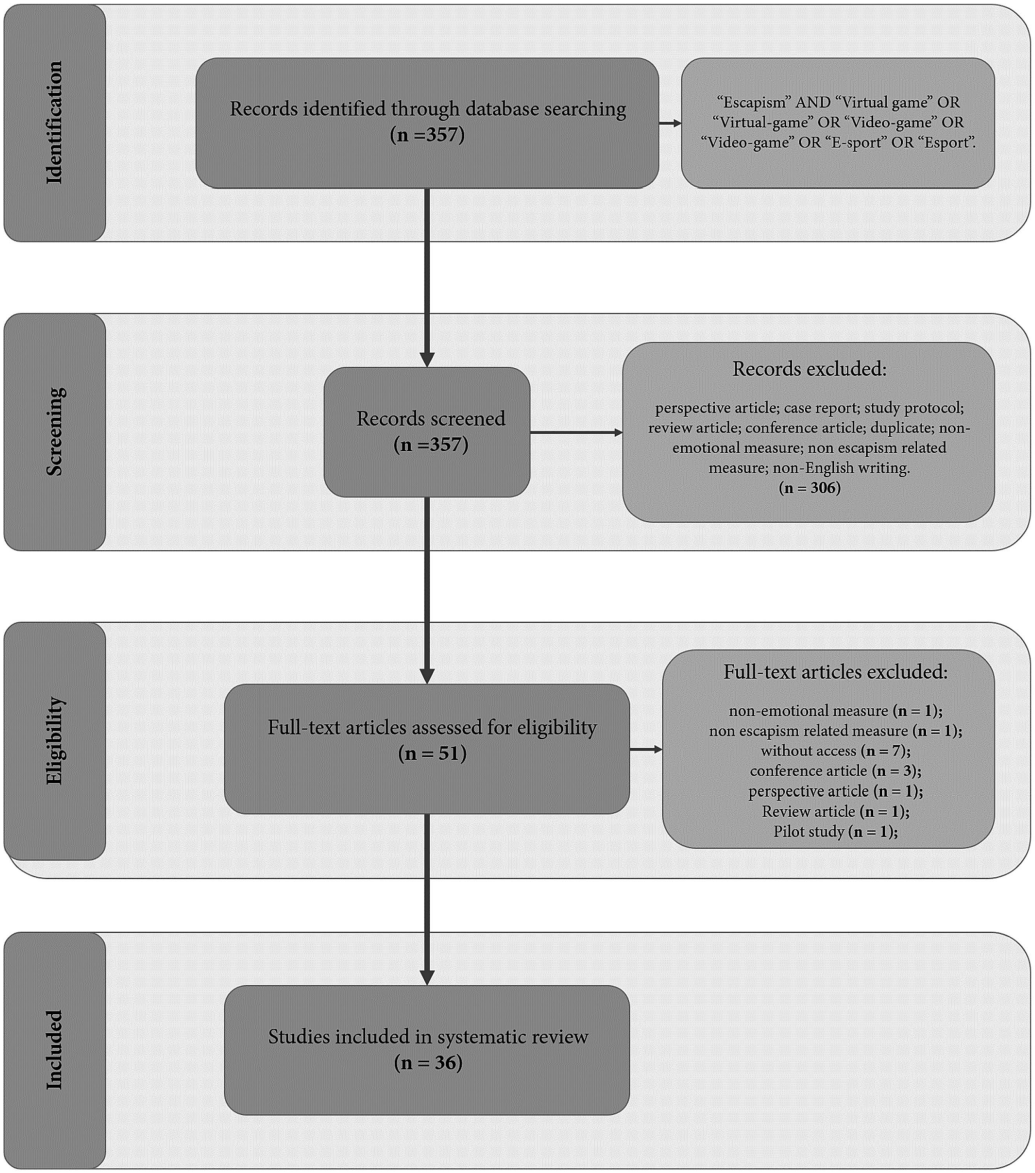
Flow diagram.

The outcomes of the search strategy are succinctly presented in Figure 1, adhering to the PRISMA statement flow diagram guidelines (27). Following the literature search, a total of 357 articles were initially identified. Through a careful screening of titles and abstracts, 306 articles were subsequently excluded. The remaining 51 articles underwent a thorough evaluation by reading their full texts. During this phase, 15 articles were further excluded as they failed to meet one or more of the specified criteria, namely: (i) utilizing non-emotional/social/mental health related measures (one article); (ii) employing non escapism-related measures (one article); (iii) lacking access to the article (seven articles); (iv) classified as a conference article (three articles); (v) classified as a perspective article (one article); (vi) classified as a review article (one article); (vii) classified as a pilot study (one article). Ultimately, a total of 36 articles met the inclusion criteria and were included in the analysis (refer to Table 1 for details).

**Table 1:** Data extraction.

Authors	Country	Sample Size	Group	Study Design	Escapism-related measure	Emotional/mental health-related measure	Observed effect
Bowditch et al. (10)	Australia	217	157 female (72.4%), 58 male (26.9%), 1 other/prefer not to say (0.5%), and 1 (0.5%) who failed to answer.	Cross-sectional	Motivations to Play in Online Games (MPOG).	Open questions.	Escapism was associated with negative gaming outcomes.
Lin et al. (11)	Taiwan	207	Female (*n* = 45, IGD = 15, No IGD = 30), Male (*n* = 162, IGD = 54, No IGD = 58)	Cross-sectional	Open questions.	Brief Coping Orientations to Problems Experienced (COPE); Coping by Gaming Questionnaire (CGQ); Resilience Scale (RS); Perceived Stress Scale (PSS); Center for Epidemiological Studies’ Depression Scale (CES-D).	Escapist motivation is the most significant motivational predictor of IGD.
Peeters et al. (28)	Netherlands	3,348	T1 (education: 50% lower, 25% middle, and 25% high), The mean age was 13.3 (SD = 0.91), and 57% were boys at T1. T2 (74%; education: 54% lower, 31% middle, and 15% high).	Cross-sectional	Open questions.	Open questions.	Escapism symptoms might indicate underlying problems, but not problematic gaming.
Tang et al. (9)	Germany	1,502	male (*n* = 805; 53.6%), female (*n* = 697; 46.4%), age: 14–39	Cross-sectional	Open questions.	Internet Gaming Disorder Scale–Short-Form (IGDS9-SF).	Escapist motivation fully mediated association between Narcissism and Psychopathy and partially mediated Machiavellianism and psychopathy.
Chen and Chang (12)	Taiwan	508	58.8% were male and 41.7% were female, and their ages ranged from 16 to 47 years	Cross-sectional	Motivations of Play in Online Games (MPOG).	Open questions.	Escapist motivation significantly moderates loneliness and negative outcomes.
Blasi et al. (13)	Italy	390	Participants’ ages ranged from 18 to 67 years (*M* = 28.28,SD = 8.24),and the sample was predominantly composed of men (*n* = 289, 74.10%).	Cross-sectional	Motives for Online Gaming Questionnaire (MOGQ).	Difficulties in Emotion Regulation Scale (DERS-18).	Escapist motivation in Emotion dysregulation individuals may strengthen PG.
Biolcati et al. (14)	Italy	202	124 (61.4%) men, aged between 18 and 48 years (*M* = 27.85, SD = 6.49). With respect to the educational level, 9.4% (*n* = 19) of the study sample had a junior high school certificate, 52.5% (*n* = 106) had a high school diploma, 22.3% (*n* = 45) had a bachelor’s degree, 11.9% (*n* = 24) had a master’s degree, and 4.0% (*n* = 8) had a specialist certificate or PhD.	Cross-sectional	Motives for Online Gaming Questionnaire (MOGQ).	Internet Gaming Disorder Scale-Short Form (IGDS9-SF).	Escapist motivation and gender were the strongest risk factors for IGD.
Deleuze et al. (29)	Belgium, Italy, United Kingdom, Luxembourg and Switzerland	273	273 participants (222 males) aged between 18 and 34 years (*M* = 21.54, SD = 3.02), who indicated playing between 1 and 40 h per week (*M* = 15.86, SD = 9.16)	Cross-sectional	Motives for Online Gaming Questionnaire (MOGQ).	Addiction-Engagement Questionnaire (AEQ)	Escapist motivation individuals had a more pronounced positive implicit attitude toward virtual environments.
Zanetta Dauriat et al. (7)	Switzerland	696	696 respondents (648 males). The mean age was 25.83 years (SD = 7.36, range: 13–54)	Cross-sectional	Open questions.	Interview.	Escapist motivation significantly predicted game addiction.
Okazaki (30)	Japan	164	Our study targeted respondents who were first- and second-year business major students from the Department of Finance and Marketing Research at Universidad Autónoma de Madrid, Madrid, Spain, as well as students from five large universities in the greater Tokyo area. Of the respondents, 78% were male, 22% were female, and 95% fell within the age range of 18 to 25 years old.	Cross-sectional	Open questions.	Interview.	Escapism as a measure of experiential value for the disposition to online mobile gaming.
Jouhki (20)	Finland	1,022	The study participants were Finnish residents aged 18–75 years (*n* = 1,530) who responded to a longitudinal survey conducted in three parts. Data were collected for the first time point (T1) in April 2021; the first follow-up survey (at Time 2, T2) took place in October–November 2021, with a response rate of 78% (*n* = 1,200), and the second follow-up survey (at Time 3, T3) took place in April–May 2022, with a 72% response rate versus T2 (*n* = 1,100). In total, 1,022 respondents took part in all three waves, representing 66.80% of the original T1 respondents.	Cross-sectional	Motivations to Play in Online Games (MPOG).	Compulsive Internet Use Scale (CIUS); Internet Gaming Disorder Test (IGDT); Problem Gambling Severity Index (PGSI).	Escapist motivation had strong and independent within-person effects on excessive gambling, excessive gaming, and excessive internet use.
Bonner (31)	Australia	186	Healthy individuals (81.8% male) aged 18–58 (*M* = 24.1)	Cross-sectional	Motives for Online Gaming Questionnaire (MOGQ).	Metacognitions about Desire Thinking Questionnaire (MDTQ).	Escapist motivation is positively associated with positive metacognitions about desire thinking.
Maroney (22)	Australia	2,261	Healthy individuals (88.7% male) aged 18–64 (*M* = 23.7).	Cross-sectional	Motivations to Play in Online Games (MPOG).	The Problematic Video Game Playing Test (PVGT); Depression Subscale of The Depression, Anxiety and Stress Scale (DASS-21); Social Interaction Anxiety Scale (SIAS); and Social Phobia Scale (SPS); The University of California, Los Angeles Loneliness Scale Revised (ULCA); The Video Game Uses and Gratification Instrument (VGUGS).	Escapist motivation positively predicts all measures related to stress; Escapism significantly mediated the relationship between social anxiety and problems with virtual game use.
Cairns (32)	UK, USA and Canada	194	Healthy players (age: *M* = 32; SD 7.8), and disabled players (age: *M* = 24; SD 8.2)	Cross-sectional	Interview.	Interview.	Players with disabilities describe the gaming experience as an effective way to escape the difficulties arising from their disability.
Fuster (33)	Spain	430	MMORPG players (45.1% males) aged 16–45 (*M* = 26.7)	Cross-sectional	Motivations to Play in Online Games (MPOG).	Interview.	Escapist motivation as one of the reasons to play MMORPG.
Kaczmarek (34)	Poland	1,056	healthy gamers (93.3% male) aged 12–49 (*M* = 18.6)	Cross-sectional	Motivations to Play in Online Games (MPOG).	Steen Happiness Index (SHI); Perceived Game Realism Scale (PGRS).	Escapist motivation was positively associated with game realism beliefs and time playing MMORPGs.
Billieux (21)	Belgium, France and Switzerland	690	World of Warcraft players (male 87.1%) aged 18–66 years (*M* = 26.2)	Prospective observational study	Motivations to Play in Online Games (MPOG).	Internet Addiction Test (IAT).	Escapist motivation was positively associated with playing time and addictive gaming behavior.
Liao (35)	Taiwan	1785	Online players (male 77.8%), aged 19–30 years	Cross-sectional	Open questions.	Open questions.	Escapism was positively predicted by autonomy and competence frustrations; Escapism significantly mediates real-world frustration and continued playing intention.
Pyszkowska (36)	Poland	189	Adult players with autism spectrum conditions (male = 26.4%), aged (*M* = 27.5)	Cross-sectional	Gaming Motivation Scale (GAMS).	Autistic Burnout Scale (AASPIRE); Positive and Negative Affect Schedule (PANAS); Temporal Experience of Pleasure Scale (TEPS).	Self-suppression Escapism was predicted by introjected regulation, positive and negative affect, and hedonic tone; Self-expansion Escapism was predicted by identified and integrated gaming motivations, hedonic tone, and positive affect.
Hagström (37)	Sweden	201	Forum users; (Male 91.5%), age (*M* = 22.6 years) (SD = 7.99).	Cross-sectional	Motivations To Play Inventory (MTPI).	Satisfaction With Life Scale (SWLS); A short version of the Clinical Outcomes in Routine Evaluation-Outcome Measure (CORE-OM).	Escapist motivation had a stronger relationship to symptoms of Internet addiction, psychological distress, and life satisfaction than other variables and other more positive motivations to play.
Kuss et al. (38)	Netherlands	265	175 players; 189 males and 76 females with an average age of 21 years (SD = 6.5 years)	Cross-sectional	Gaming Motivation Scale (GAMS).	The Problem Video Game Playing Questionnaire (PVP).	Escapist motivation serves as a reliable predictor of problematic gaming.
Bányai et al. (23)	Hungary	4,284	Hungarian-speaking gamers; (Male = 89.89%); Age (*M* = 23.08) (SD = 6.57) (range 14–58)	Cross-sectional	Motives for Online Gaming Questionnaire (MOGQ).	Brief Symptom Inventory (BSI); Gaming Disorder Test (IGDT-10).	Escapist motivation significantly mediated psychiatric distress and IGD.
Šporčić and Glavak-Tkalić (39)	Croatia	509	Young adults (*M* = 23.14, SD = 4.66) who are virtual game players from Croatia.	Cross-sectional	Motives for Online Gaming Questionnaire (MOGQ).	Internet Gaming Disorder Scale (IGDS9); Self-Concept Clarity Scale (SCC).	Escapist motivation significantly mediated self-concept clarity and PG.
Pontes et al. (24)	USA, India and UK	1,004	English-speaking gamers from the USA, India, and the UK were recruited online on gaming forums. American (*N* = 405, minimum age = 16 years, maximum age = 70 years, 62% males). Indian (*N* = 336, minimum age = 16 years, maximum age = 69 years, 67.6% males). British (*N* = 272, minimum age = 16 years, maximum age = 70 years, 50.7% males)	Cross-sectional	Internet Gaming Disorder Scale–Short-Form (IGDS9-SF).	Internet Gaming Disorder Scale–Short-Form (IGDS9-SF).	Escapism was significantly associated with daily activities’ impairment related to gaming.
Park et al. (40)	South Korea	524	College students, who make up a significant portion of online gamers in Korea (47.6% were male; 42.3% were 20 or under, 43.9% were between 21 and 24 years, and 13.8% were 25 or over).	Cross-sectional	Open questions.	Ten Item Personality Inventory (TIPI).	Escapist motivation was significantly associated with online game playing.
Zaib Abbasi et al. (41)	Malaysia	290	Students from three Malaysian universities (participants enrolled in foundational and undergraduate degree programs, aged 18–25 years, actively engaged in esports gaming brands, and playing at least on a weekly basis).	Cross-sectional	Open questions.	Open questions.	Escapist motivation was not associated with esports engagement.
Li et al. (42)	Singapore	161	Students who reported having MMO gaming experiences from two Singapore secondary schools	Cross-sectional	Open questions.	Open questions.	Escapist motivation was significantly associated with PG.
Lee et al. (43)	USA	324	College students at a large Midwestern university in the United States (66 percent reported having played SNG before; 57.5 percent were men; the average age was 20.77, ranging from 17 to 33).	Cross-sectional	Open questions.	Open questions.	Escapist motivation was significantly associated with self-expression and the pursuit to make good impressions on others.
Engelhardt et al. (44)	USA	119	119 adults (16 women), half of whom had a previous diagnosis of ASD, while the other half were typically developing (TD) adults. ASD age 20.4, TD age 20.5	Prospective observational study	Open questions.	Open questions.	Escapist motivation was significantly higher in the ASD group when compared to the control group.
Frostling-Henningsson (45)	Sweden	23	Young adults who are online gamers.	Cross-sectional	Interview.	Interview.	Escapism motivated by social reasons provides gamers with an experience in which they can attain a state of “flow” and serves as a “hallucination of the real.”
Alimamy et al. (46)	United Arab Emirates and Canada	300	Participants ranged in age from 18 to 74 years old.	Cross-sectional	Interview.	Interview.	Escapist motivation significantly predicts mental health.
Wischert-Zielke and Barke (47)	Germany	2,909	Internet Gaming Disorder (IGD), Women: 2725; men: 135; non-binary: 49 – mean age of 31.1 ± 8.9 years	Cross-sectional	Motives for Online Gaming Questionnaire (MOGQ).	Brief Symptom Inventory (BSI-18).	Escapist motivation was significantly associated with IGD.
McKenna et al. (48)	USA	10	Mean age of 16.60 years.	Cross-sectional	Interview.	Transgender Congruence Scale (TCS).	Escapism through online gaming and virtual game avatar can promote gender-affirmation and exploration in Transgender and gender diverse adolescents.
Lee and Chen (49)	USA	480	Participants who were unemployed and resided within the United States (age mean 34.23 years old, female (*n* = 244) male (*n* = 152))	Cross-sectional	Open questions.	Open questions.	Escapism through virtual games promotes the sense of control in unemployed workers.
Sauter et al. (50)	109 different countries	13,464	Healthy individuals	Cross-sectional	Open questions.	Satisfaction With Life Scale (SWLS); General Anxiety Disorder-7 (GAD-7); Social Phobia Scale (SPS).	Escapist motivation significantly predicts mental health.
Boldi et al. (51)	Italy	330	Players; (257 males, 69 females, 4 non-binary); Age (range from 18 – more than 65) (37.9% between 27 and 35 years old)	Cross-sectional	Motivations To Play Inventory (MTPI).	Open questions.	Escapist motivation could yield a disruption of temporal routines, perception of drain of energy, damage in household relationship, and increase players’ sedentariness.

### Demographic findings

Among the extracted information in the present study, there were 3 data related to demographic findings, such as: (i) country; (ii) sample size; (iii) which group characterized the study sample.

#### Country

In reference to the focal country of the study, we observe:

Australia – three articles (10, 22, 31); Croatia – one article (39); Finland – one article (20); Germany – two articles (9, 47); Hungary – one article (23); Italy – three articles (13, 14, 51); Japan – one article (30); Malaysia – one article (41); Netherlands – two articles (28, 38); Poland – two articles (34, 36); Singapore – one article (42); South Korea – one article (40); Spain – one article (33); Sweden – two articles (37, 45); Switzerland – one article (7); Taiwan – three articles (11, 12, 52); The United States of America – four articles (43, 44, 48, 49); Multiple countries – five articles (21, 24, 29, 46, 50).

Furthermore, it is important to emphasize the fact that some of the articles obtained data via internet questionnaires, thus collecting data beyond their intended country.

#### Sample size

Regarding the sample size, the articles presented a total sample of 40,514 participants (*M* = 1,095,1; SD = 2,333.8; minimum = 10, maximum = 13,464). The characteristics of the reported samples were collected based on three categories: gender, age, and center characteristics. Regarding gender, the total participants showed a predominance of male individuals (72.2%), with females accounting for only 23.7% of the total participants in the study, while others (4.1%) could not access the data or identified with another gender.

#### Age

In the age category, we distributed all studies into 4 groups: (i) Children (0–12 years) (ii) Adolescents (12–18 years) (iii) Adults (18–65 years) (iv) Older adults (older than 65 years). Thus, we observed the following distribution of articles: Adolescents – three articles (28, 42, 48); Adolescents and adults – eleven articles (7, 9, 12, 23, 33, 34, 37, 38, 40, 43, 45); Adolescents, adults and older adults – one article (24); Adults – fifteen articles (10, 11, 14, 22, 29–32, 36, 39, 41, 44, 47, 49, 52); Adults and older adults – six articles (13, 20, 21, 46, 50, 51). Most studies included adult individuals. Only three studies focused exclusively on adolescents, and all the studies with older adults were conducted in conjunction with adults.

#### Sample group

Finally, regarding the center characteristics, we observed: Autism spectrum disorder – two articles (36, 44); Internet Gaming Disorder – three articles (11, 28, 47); Healthy individuals – three articles (22, 31, 50); MMO players – one article (42); MMORPG players – seven articles (10, 13, 14, 21, 33, 37, 38); Recreational and e-sport players – one article (23); First person shooter players and MMORPG players – one article (45); Nonspecific players: nine articles (7, 24, 29, 32, 34, 39, 46, 51, 52); Users of virtual game live streaming services – one article (12); German internet users – one article (9); Transgender and gender-diverse – one article (48); United State residents – one article (49); Finnish residents – one article (20); University students – two articles (30, 41); College Students – two articles (40, 43).

### Study design

Out of the 36 articles reviewed in the present study, 34 employed a cross-sectional study design, while the remaining 2 utilized prospective observational study designs.

### Escapism-related measure

Among the studies analyzed, it was possible to observe that the primary measure of escapism was often the assessment of motivation for gaming—specifically, as a means to escape reality. These articles typically evaluated Escapist Motivation (EM) alongside other motivations such as coping, fantasy, skill development, recreation, competition, and social interactions. Some studies also explored escapism through open-ended interviews and specific questions dedicated to the concept of escapism.

As presented in Table 1, we have extracted information corresponding to each specific instrument. The breakdown of measures related to escapism is as follows: Gaming Motivation Scale (GAMS) – two studies (36, 38); Internet Gaming Disorder Scale–Short-Form (IGDS9-SF) – one study (24); Interview – four studies (32, 45, 46, 48); Motivations of Play in Online Games (MPOG) – seven studies (10, 12, 20–22, 33, 34); Motivations To Play Inventory (MTPI) – two studies (37, 51); Motives for Online Gaming Questionnaire (MOGQ) – seven studies (13, 14, 23, 29, 31, 39, 47); Open questions – 11 studies (7, 9, 28, 30, 40–44, 49, 52).

From these results, it is evident that most studies employed instruments that have been validated in existing literature, thereby enhancing the credibility of the findings. However, a notable number relied on open-ended questions and interviews. While this methodological choice provides a more comprehensive view of the phenomenon, it lacks the precision of structured instruments. Importantly, all the tools used in these studies are predicated on participants’ subjective evaluations concerning their tendencies toward escapism. They do not act as experimental measures that directly assess the escapism behaviors exhibited by participants. Such subjective evaluations might be influenced by the participants’ intentions to manage or shape the perception held by evaluators.

### Emotional/social/mental health-related measure

Concerning the measures of emotional, social, and mental health employed to investigate potential associations with escapism behavior, an examination of Table 1 and the subsequent list reveals the utilization of 36 distinct instruments. Beyond these, four studies incorporated open interviews, and nine deployed open-ended questions specifically targeting escapism. In the sections that follow, we delineate each instrument and cite the respective articles that employed them.

Autistic Burnout Scale (AASPIRE) – one study (36); Addiction-Engagement Questionnaire (AEQ) – one study (29); Brief Symptom Inventory (BSI) – two studies (23, 47); Brief Symptom Inventory (BSI-18) – one study (47); Center for Epidemiological Studies’ Depression Scale (CES-D) – one study (11); Coping by Gaming Questionnaire (CGQ) – one study (11); Compulsive Internet Use Scale (CIUS) – one study (20); Coping Orientations to Problems Experienced (COPE) – one study (11); Clinical Outcomes in Routine Evaluation-Outcome Measure (CORE-OM) – one study (37); Depression Subscale of The Depression, Anxiety and Stress Scale (DASS-21) – one study (22); Difficulties in Emotion Regulation Scale (DERS-18) – one study (13); General Anxiety Disorder-7 (GAD-7) – one study (50); Internet Addiction Test (IAT) – one study (21); Internet Gaming Disorder (IGD) – one study (20); Internet Gaming Disorder Scale (IGDS9) – one study (39); Internet Gaming Disorder Scale–Short-Form (IGDS9-SF) – three studies (9, 14, 24); Internet Gaming Disorder Test (IGDT) – two studies (20, 23); Interview – four studies (32, 45, 46, 48); Gaming Disorder Test (IGDT-10) – one study (23); Metacognitions about Desire Thinking Questionnaire (MDTQ) – one study (31); Positive and Negative Affect Schedule (PANAS) – one study (36); Problematic Gaming (PG) – one study (20); Perceived Game Realism Scale (PGRS) – one study (34); Problem Gambling Severity Index (PGSI) – one study (20); Perceived Stress Scale (PSS) – one study (11); The Problematic Video Game Playing Test (PVGT) – one study (22); The Problem Video Game Playing Questionnaire (PVP) – one study (38); Resilience Scale (RS) – five studies (11, 13, 22, 37, 40); Self-Concept Clarity Scale (SCC) – one study (39); Steen Happiness Index (SHI) – one study (34); Social Interaction Anxiety Scale (SIAS) – one study (22); Social Phobia Scale (SPS) – two studies (22, 50); Satisfaction With Life Scale (SWLS) – two studies (37, 50); Transgender Congruence Scale (TCS) – one study (48); Temporal Experience of Pleasure Scale (TEPS) – one study (36); Ten Item Personality Inventory (TIPI) – one study (40); Los Angeles Loneliness Scale Revised (ULCA) – one study (22); The Video Game Uses and Gratification Instrument (VGUGS) – one study (22); and Open questions – nine studies (10, 12, 28, 41–44, 49, 51).

Predominantly, the studies under review assess dimensions pertaining to emotional and psychological health, psychiatric evaluations, social well-being, and problematic gaming. As delineated in the preceding section, measures associated with emotional, social, and mental health are largely predicated on subjective self-assessment. Consequently, these instruments do not capture clinical perceptions nor do they provide an objective quantification of the emotional state, such as would be garnered through physiological tools or implicit association tasks. Nevertheless, aside from those studies employing interviews and singular questions, all others have harnessed structured and validated instruments to gauge the specific phenomena under investigation.

### Observed effect

Subsequent to detailing the instruments related to escapism and emotional, social, and mental health, we collated the findings from the 37 studies. As delineated in Table 1, a myriad of results emerged, each unique to its respective study, necessitating the categorization of results into clusters. All authors initially undertook separate analyses, which were subsequently synthesized to formulate cohesive groupings of results.

**Figure 2:**
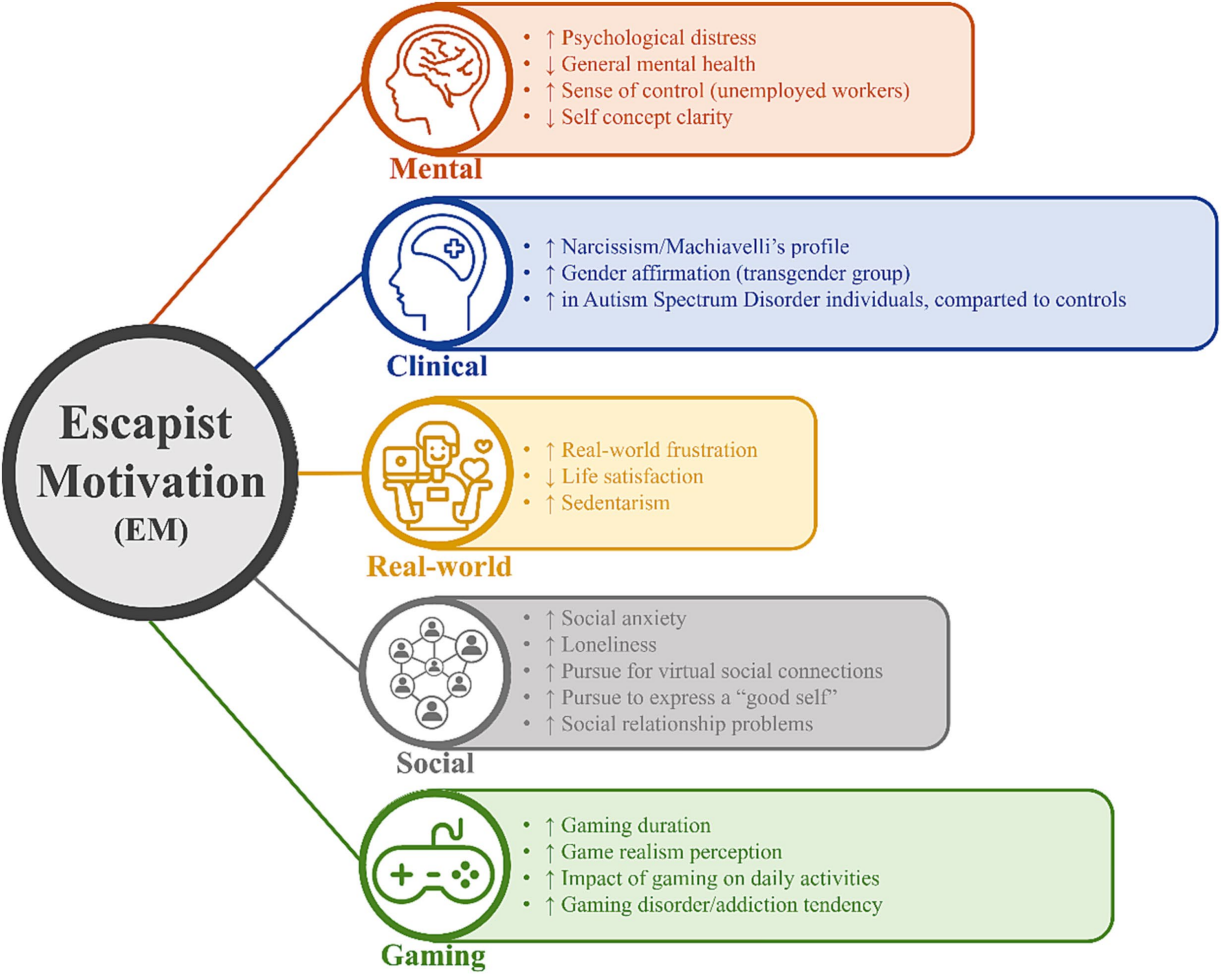
Main aspects associated with Escapist Motivation (EM). The character “↑” means positive association with EM, while “↓” means negative association with EM.

The subsequent paragraphs delineate the five distinct clusters, comprising 20 groups of findings, each associated with specific articles. Additionally, Figure 2 provides a comprehensive summary of these five clusters and their respective 20 groups of findings. It is worth mentioning that five distinct clusters were formulated, organizing the 20 findings within a theoretical framework. While it is conceivable that a finding might fit into multiple clusters, this configuration was chosen to enhance clarity and facilitate a logical comprehension of the findings. In the sections that follow, each cluster is delineated individually, accompanied by a succinct introduction explaining its thematic grouping.

#### Mental

The Mental cluster grouped results related to aspects of psychological well-being, mental health, and psychological processes of self-assessment. The main groups of results were: (i) EM positively associated with psychological distress – three articles (22, 23, 37); (ii) EM positively associated with general mental health – one article (50); (iii) EM positively associated with sense of control (unemployed workers) – one article (49); and (iv) EM negatively associated with self-concept clarity – one article (39).

In general, it is possible to understand that EM is negatively associated with aspects of good emotional/mental-health; except for the finding that EM is positively associated with a sense of control among unemployed workers (49).

#### Clinical

The Clinical cluster aggregated findings pertinent to characteristics associated with clinical cohorts, including: (i) EM positively associated with Narcissism/Machiavelli’s profile – one article (9); (ii) EM positively associated with gender affirmation (transgender group) – one article (48); and (iii) Autism Spectrum Disorder individual present higher EM compared to controls – one article (44).

Predominantly, the evidence indicates that Escapist Motivation (EM) correlates with clinical characteristics detrimental to optimal social and emotional adaptation. An exception is observed in transgender individuals who, driven by the desire to escape reality, achieve gender affirmation and representation within games via their virtual avatars (44).

#### Real-world

The ‘Real-world’ cluster collated findings pertinent to an individual’s perception of their immediate environment, introspective reflections on life, and strategies toward physical well-being. The results observed were: (i) EM positively associated with real-world frustration – two articles (32, 35); EM negatively associated with life satisfaction – two articles (34, 37); EM positively associated with sedentarism – one article (51).

Accordingly, the findings suggest that Escapist Motivation (EM) is positively correlated with a diminished subjective perception of one’s life, their surrounding environment, and personal self-care.

#### Social

The ‘Social’ cluster integrated findings pertinent to an individual’s pursuit of new social affiliations, the sustenance of existing relationships, and the cultivation of a favorable perception of others. This also encompasses facets like social anxiety and perceptions of solitude. The main groups of results were: (i) EM positively associated with social anxiety – one article (22); (ii) EM positively associated with loneliness – one article (43); (iii) EM positively associated with pursue for virtual social connections – one article (45); (iv) EM positively associated with pursue to express a “good self” – one article (12); and (v) EM positively associated with social relationship problems – one article (51).

In summary, the results indicate that EM correlates with suboptimal social adaptation. Despite being associated with the pursuit of new virtual social affiliations, it can be inferred that this virtual engagement may signify an alienation from real-world connections.

#### Gaming

Finally, concerning the ‘Gaming’ cluster, findings pertaining to gameplay, perception of this practice, and its adverse outcomes were categorized. The data reveal that (i) EM positively associated with gaming duration – two articles (21, 34); (ii) EM positively associated with game realism perception – one article (34); (iii) EM positively associated with the impact of gaming on daily activities – eight articles (10, 13, 20, 24, 38, 39, 42, 51); (iv) EM positively associated with gaming disorder/addiction tendency – six articles (11, 14, 21, 23, 37, 47); and (v) EM is not a predictor of gaming engagement in Esports players – one article (41).

In synthesis, the findings indicate that EM is positively associated with an unhealthy practice, with excessive playing time, clinical addiction to games and skewed perceptions of gaming. It is worth mentioning the result that EM is not associated with game engagement in esports practitioners (41). This may hint that their motivation stems from other elements, including skill advancement, competitiveness, and leisure.

## Discussion

This review aimed to elucidate the relationship between escapist behavior in virtual games and mental health. The compilation of 36 articles predominantly utilized measures of Escapist Motivation (EM) to identify significant correlations between EM and various facets of mental health, categorized into five distinct clusters. Based on the aggregated results, it can be inferred that EM has a negative correlation with mental health outcomes, whether these pertain to gaming habits, emotional processes, ramifications on social interactions, or the nexus between real-life and virtual experiences. Nonetheless, it is worth mentioning that all findings from the reviewed articles are based on participants’ subjective self-assessments regarding their motivations to seek escape through virtual gaming. No experimental evaluations were conducted in any of the articles to empirically verify escapism behavior. Consequently, the results should be interpreted with circumspection as they represent self-reported Escapist Motivation (EM) rather than observed escapism behavior.

In the subsequent sections, we delve into the multifaceted aspects of EM. We commence with an exploration of escapism as a primary motivation for engaging in virtual games. This is followed by discussions on the association of EM with gaming practices, mental health, social well-being, and perceptions of real-world experiences. We conclude by examining the implications of escapist behavior for health promotion, public health, and clinical practice This last topic is directly aligned with the theme of this special issue in which our work is inserted.

### Escapism as a motive/motivator

Predominantly, research has shown that escapism is one of the main reasons for playing virtual games. Moreover, as discerned from this review, escapism can potentially compromise a player’s health and act as a mechanism detracting from social and emotional adaptation. Some articles have observed a relationship between escapism and social anxiety (12, 50) and loneliness (12, 22). In multiplayer online role-playing games, these effects become more pronounced, leading to an escalation in anxiety over time when the player engages with unfamiliar participants (50). Conversely, engaging in games with acquaintances from one’s real-life social circle can enhance a player’s life satisfaction and reduce their vulnerability to social anxiety (50). When comparing Massively Multiplayer Online Role-Playing Game (MMORPG) players with First Person Shooter (FPS) players, it was observed that MMORPG players have stronger motivations for social interaction, while FPS players reported stronger motivations for escapism (22). Additionally, MMORPG players apparently play for longer periods of time (22), perhaps due to the need for social interaction.

In our review, it was highlighted that escapism is primarily gaged using instruments assessing motivations for virtual game engagement. Not only do these studies determine if participants seek escape, but these subjective tools also report escapism as a significant reason for playing. Such self-assessments do not provide clinical insights or objective measurements of emotional states, yet they underline the escapism motive, linking it to adverse outcomes. It is imperative to differentiate between evaluating the mere effect of escapism and the underlying motivation behind it. Ultimately, the best way to determine when the practice of virtual gaming can pose a risk or benefit to mental health is by evaluating the motive or motivator.

It is important to discuss other motivations and compare them to escapism. Zanetta et al. (7) presents an extensive discussion on game motivations, proposing 10 motivations and linking them to 39 items using a factor loading scale. The 10 motivations and their strongest relationships are as follows: (i) *Advancement*: Becoming powerful; (ii) *Mechanics*: How interested are you in the precise numbers and percentages underlying the game mechanics?; (iii) *Competition*: Doing things that annoy other players; (iv) *Socializing*: Getting to know other players; (v) *Relationship*: How often do you talk to your online friends about your personal issues?; (vi) *Teamwork*: Would you rather be grouped or soloing?; (vii) *Discovery*: How much do you enjoy exploring the world just for the sake of exploring it?; (viii) *Role-Playing*: How often do you role-play your character?; (ix) *Customization*: How important is it to you that your character’s armor/outfit matches in color and style?; and (x) *Escapism*: Escaping from the real world. It is mentioned that “Several players described how these online environments provided social outlets to which they do not have access in real life. For them, MMORPGs served a much-needed social function,” indicating that “escapism” appears to be the motivator associated with negative outcomes.

### Gaming practice

Considering that an important concern regarding the practice of virtual games is the development of IGD, given the results found in the present review, it is worth highlighting the association between IGD and EM.

It has been observed that Escapist Motivation is the strongest predictor of Internet Gaming Disorder (IGD), contributing with loss of control over gaming and the time spent on it (21, 34, 52), as well as increasing the priority of gaming upon other activities (10, 13, 20, 24, 38, 39, 42, 51). Looking deeper into Escapist Motivation, it is known that different motives for escaping in gaming can lead to various outcomes when it comes to gaming practice and its characteristics (11, 14, 39, 47).

While numerous factors independently predict, they gradually converge as prominent motivations for gaming, ultimately manifesting as a form of escapism. Understanding this correlation allows us to assert that factors such as autonomy, frustration, coping, competition, social motives, and fantasy pursuit can predict IGD because they are considered to be causes of Escapist Motivation (52). Individuals who feel the need to escape often turn to gaming as an option, and while this is evident, it is equally important to delve into the impact that escapism has on gaming practice characteristics. In place of regular gaming, individuals with a strong inclination for escapism are at a higher risk of developing addictive gaming habits. In such instances, gaming often takes precedence as a central activity in the individual’s life, displacing valuable time that could have been allocated to other essential life activities had problematic gaming not been a factor.

The amount of time spent on gaming activities is correlated to either online and offline social support. Increased gaming time has been observed as a predictor of lower offline social support, while being related to increased online social support from other players. Offline social support is known to promote well-being, but in certain cases in which this type of social support is not present or sufficient, online social support appears as an alternative to compensate for decreased well-being (34). Thus, individuals’ search for well-being increases gaming time.

Apart from recreational and conventional gaming, there is another gaming genre: competitive Esports. Much can be discussed about the difference between gaming for leisure and gaming as a job, but although “enjoyment, sensory experiences, emotional involvement, and arousal positively affect consumers’ esports game engagement,” research has not been able to show whether EM, role-projection and fantasy are or are not related to esports engagement (41).

### Mental health

Escapism seems to mediate the relationship between real world problems and virtual game use, thus being intrinsically correlated with measures related to stress, psychological distress, mental health, and life satisfaction (22, 23, 37, 50). This supports the idea that individuals with greater difficulty adapting to the real world often retreat into games, thus also correlating with narcissistic individuals, transgender individuals, and autism (9, 44, 48).

This relationship of poorer mental health with virtual games escapism seems to be more related to MMORPG and First-person shooter games, due to their power to escape from aversive states (22). This corroborates the research where transgender people tend to play RPG model games due to their possibility to express themselves as avatars (48). This also resonates with another observed article, showing a correlation between virtual games and a sense of control in unemployed workers, as one of the main reasons for individuals to escape from reality to the world of virtual games (49).

One detrimental aspect of EM seems to be the potential decline in self-concept clarity (48). This lack of clarity may result in individuals failing to develop effective emotional coping strategies for dealing with adverse situations in the real world, leading them to seek refuge in the realm of video games This escape does not offer genuine solutions to real-life challenges and may diminish their connection to essential social support systems due to time spent on games (22), thus intensifying psychological problems such as depression.

It is worth highlighting the lack of longitudinal studies; only one study is not cross-sectional (44). Therefore, there is a gap in understanding how these problems develop and impact mental health in the long or short term. Moreover, there is a dearth of research on the relationship between escapism and other psychological disorders, such as depression., as some studies have explored the connection between escapism and mental health, but often the participants in these studies are either generally healthy or lack confirmed diagnoses of specific psychological disorders, which can limit the broader understanding of how escapism may interact with various mental health conditions., as in the case of the studies (22, 23, 37, 44, 50).

### Social health

Social health plays a pivotal role in shaping individuals’ motivations for engaging in virtual games. People with social anxiety usually have two contrasting motives: playing as a means of social interaction or playing as a form of EM. Games can assist individuals with social anxiety to better interact through the characters and story of the game. On the other hand, social anxiety could cause stress and motivate a person to play in order to avoid the judgment of other people (22). The motivation behind gaming exerts a substantial influence on several key factors, including the amount of time dedicated to gaming, the specific genre or type of game chosen, as well as an individual’s mental health and the presence of social anxiety issues. Generally, EM is associated with more severe social anxiety, playing first-person games (FPS), and spending less time playing compared to those who play for social interaction (22). Thus, the motive behind someone’s gaming habits is a crucial determinant in assessing and understanding the impact on the player’s health.

The act of playing virtual games can be erroneously associated with a solitary lifestyle. By considering various degrees of escapism, the conventional belief that psychologically and socially well-adjusted individuals derive benefits from engaging in virtual games, whereas those who are not in good health do not, is deconstructed. It becomes apparent that individuals who lead solitary lives can derive greater advantages from frequent involvement in gaming compared to their similarly isolated counterparts who engage less frequently. This phenomenon arises from the ability of virtual games to create an immersive “world” wherein individuals can experience a sense of inclusion and interact with diverse individuals. Consequently, escapism serves as a moderator of the sensation of loneliness, enabling the promotion of player immersion and compensating for the dearth of real-world interactions.

The transformations brought about by the COVID-19 pandemic in the social life of the entire world are undeniably significant. So profound are these changes that a clear distinction between the pre-pandemic and post-pandemic eras is perceptible. It is impossible to disregard the importance of virtual games during the lockdown and how escapism played a vital role in maintaining social well-being. The prevailing perception is that virtual games provided an avenue for escapism through interaction among individuals who were unable to engage in face-to-face interactions due to the lockdown, and indeed, this is true. However, another crucial factor is that escapism allows virtual game players to “escape” from the mundane, monotonous, unsatisfying, and solitary reality. Escapism aided people in maintaining social well-being in various ways, both for those who were alone at home and for those who were with their families but experiencing strained relationships. The creation of new activities in a new virtual world sustained interactions and contributed to the preservation of mental health for those who had the opportunity to engage in virtual gaming.

Therefore, playing online games is not merely about the desire to play in order to isolate oneself from social interaction; rather, it represents the arise of a new form of social interaction. Even in first-person shooter (FPS) games, the sentiments of cooperation and teamwork are crucial for achieving optimal performance in the game (45). The ability to communicate during gameplay is of great importance. Communication extends beyond in-game details and serves as a pathway for players to freely express themselves, such as sharing personal experiences from their real lives as a means of unburden (45). Furthermore, there exists the possibility for individuals to “live” in games what they are unable to experience in the real world, facilitated by new avenues where one can construct, for example, a new lifestyle, a different physical appearance, gender, or even age. Undeniably, the virtual world provided by games can be interpreted as a world where people are free to escape reality and embody different behaviors from real life, in whichever way they desire. This allows for the social experience of multiple lives within a single existence, thus creating a more democratic form of social interaction.

### Real-world life

The impact of virtual gaming on real-world life can take two directions: it can be beneficial, enhancing various aspects of life, or it can move away from these potential benefits, leading to problems for gamers. Frustration in real-world life may drive gamers to spend increasingly more time gaming, causing them to become emotionally detached or absent from their own lives (52). Indeed, it can create a kind of revolving door when personal fulfillment becomes more closely linked with the virtual world, serving as an escape from negative thoughts and challenges in real life. In this scenario, individuals may become so engrossed in the virtual realm that they forget about their real-life concerns, effectively “living” in a more desirable virtual world (32).

Even though escapism from real life thought gaming may seem like an appealing option, it can have adverse effects on the well-being of individuals who are isolated users with low self-esteem (53) and for emotionally sensitive players these “virtual” places must be mediated by social spaces to prevent negative impacts on their real-world well-being (54). Moreover, when the virtual world starts to blur with reality, players may inadvertently neglect themselves, their relationships, and essential self-care activities. Additionally, there is evidence of increased dissociative experiences, such as depersonalization and derealization (55).

### Health promotion, public health, and clinical practice

Based on the findings discussed in the preceding sections, it is apparent that the practice of virtual gaming, primarily driven by escapist behavior, can have significant implications for public health. As presented in the introduction of this work, the practice of virtual gaming has become increasingly prevalent worldwide, cutting across various social classes, genders, and age groups (56). This expansion, in tandem with the gaming industry’s growth, coincides with the increasing body of research highlighting the advantages of gamification in enhancing well-being and promoting health (57–59). However, it is imperative to consider other facets of this growth, including the potential negative consequences associated with the practice of virtual gaming. These adverse effects can encompass physical health implications, along with impacts on emotional, social, and mental well-being, as well as the broader influence on social processes and even the sociocultural development (60, 61). The present work observed that the practice of virtual games driven by the primary motivation of escaping from reality can not only signal potential harm to the individual’s mental state but also indicate possible adverse effects on their socialization and overall well-being. Consequently, it becomes evident that the impact of virtual gaming on mental health is contingent upon a thorough assessment of the underlying motives and motivators driving its practice.

Broadly understanding these findings, virtual games represent a readily accessible activity, often without time restrictions and available for at-home consumption. When engaged in as a form of escapism, they have the potential to negatively affect the mental health of a significant portion of the population, although not necessarily following a clear causal pathway. It is important to note that this study does not aim to categorize gaming as a direct threat to public mental health but rather to highlight the risks associated with non-adaptive motivations, which can subtly contribute to mental health challenges. Additionally, clinical professionals should consider the aggregated findings when working with patients who engage in virtual gaming. Future research should explore these impacts more comprehensively through experimental investigations, focusing on emotional, social, mental, and real-life aspects of individuals practicing virtual games for escapism.

### Limitations

The primary limitation of this study pertains to the diverse instruments used in the research to assess potential associations between EM and emotional, social, and mental health. As evident from our results, some studies employed well-established and validated tools like the Gaming Motivation Scale (GAMS), Internet Gaming Disorder Scale–Short-Form (IGDS9-SF), Motives for Online Gaming Questionnaire (MOGQ), among others. Conversely, certain studies relied on interviews and open-ended questions to gauge EM. While interviews and open questions offer the advantage of capturing nuanced aspects that structured instruments might miss, they present challenges when it comes to integrating findings for comparison, as was done in this review. Consequently, the choice of assessment instrument may have influenced the interpretation of certain phenomena discussed in this study, potentially hindering direct comparisons across studies. Nonetheless, since our study’s primary objective was to compile and analyze findings concerning the association between EM and emotional, social, and mental health, we believe that including studies with varied methodologies, despite their inherent limitations, enriches both the quantitative and qualitative evaluation of the phenomena. Lastly, we chose not to conduct an official quality assessment due to the experience we had with an article recently published (62). The definition of quality is very heterogeneous, and achieving an assessment that takes into account the differences between papers ends up disregarding the qualities of others. Thus, to ensure the quality of the papers included in the study, we therefore chose to include only articles from reputable journals that have undergone the peer review process.

## Conclusion

Our review underscores the evidence that Escapist Motivation (EM) for virtual gaming correlates with negative and maladaptive mental health, social health, and gaming outcomes. Furthermore, while the results do not confirm a direct causal relationship between virtual gaming driven by EM and its outcomes, they were rigorously analyzed in the context of their potential implications for public health, mental health, and clinical practice, especially regarding the desire to escape real-world challenges.

## Data availability statement

The original contributions presented in the study are included in the article/Supplementary material, further inquiries can be directed to the corresponding author.

## Author contributions

LM: Supervision, Validation, Writing – original draft, Writing – review & editing, Conceptualization, Formal analysis, Methodology, Project administration. PU: Writing – original draft, Writing – review & editing. FA: Writing – original draft, Writing – review & editing. GK: Writing – original draft, Writing – review & editing. RS: Writing – original draft, Writing – review & editing. SB: Writing – original draft, Writing – review & editing.
